# Efficient information coding and degeneracy in the nervous system

**DOI:** 10.1016/j.conb.2022.102620

**Published:** 2022-08-17

**Authors:** Pavithraa Seenivasan, Rishikesh Narayanan

**Affiliations:** Cellular Neurophysiology Laboratory, Molecular Biophysics Unit, Indian Institute of Science, Bangalore, 560012, India

## Abstract

Efficient information coding (EIC) is a universal biological framework rooted in the fundamental principle that system responses should match their natural stimulus statistics for maximizing environmental information. Quantitatively assessed through information theory, such adaptation to the environment occurs at all biological levels and timescales. The context dependence of environmental stimuli and the need for stable adaptations make EIC a daunting task. We argue that biological complexity is the principal architect that subserves deft execution of stable EIC. Complexity in a system is characterized by several functionally segregated subsystems that show a high degree of functional integration when they interact with each other. Complex biological systems manifest heterogeneities and degeneracy, wherein structurally different subsystems could interact to yield the same functional outcome. We argue that complex systems offer several choices that effectively implement EIC and homeostasis for each of the different contexts encountered by the system.

## Introduction

The incredible similarity between pearl white and cotton white and the consequent inability to choose one can be discomforting. Nonetheless, an experienced painter who has been regularly exposed to the palette of colors can distinguish them unmistakably well. Such adaptation in responses dependent on the statistical prevalence of specific stimuli can be explained by a fundamental principle called efficient information coding (EIC) (see Box 1 for definitions of important terms). Across a range of neural scales and systems, EIC is accomplished by adaptively matching the response properties of the system to the natural statistics of the stimuli [1–5]. Information-theoretic analyses [6] provide a strong substrate for formalizing and assessing EIC from the perspective of maximizing stimulus information in system responses.

There are several reasons why EIC is a daunting task. First, the response properties of the system must continually match context-dependent and time-varying stimulus prevalence. Second, multiple timescales associated with various stimulus attributes underscore a need to distinguish temporary environmental fluctuations from persistent changes. Third, adaptations should maintain system stability by recruiting concomitant homeostatic processes that do not hamper EIC. Finally, it is critical to recognize that the rules governing the emergence of EIC could be distinct across scales. Despite these, there is a growing body of evidence that the nervous system robustly accomplishes EIC across all scales.

In this review, we present a unified synthesis with illustrative examples from several species and multiple scales of the nervous system to first demonstrate the ubiquity of EIC. We also build a systematic case that the complexity of the brain is pivotal in its ability to meet the formidable challenges faced in achieving multiscale EIC. Complexity in a system is characterized by several functionally segregated subsystems that manifest a high degree of functional integration when they interact with each other [17]. A characteristic feature of such complex systems is their ability to show degeneracy, whereby structurally different subsystems could interact to yield the same functional outcome [17]. Here, we postulate that degeneracy offers a substrate for simultaneously achieving EIC and homeostasis (Box 1). Our postulate follows from the several degrees of freedom available to a complex system, in terms of the disparate interactions among different subsystems that yield the same functional goal of stable EIC.

### EIC spans multiple scales

**Figure 1:**
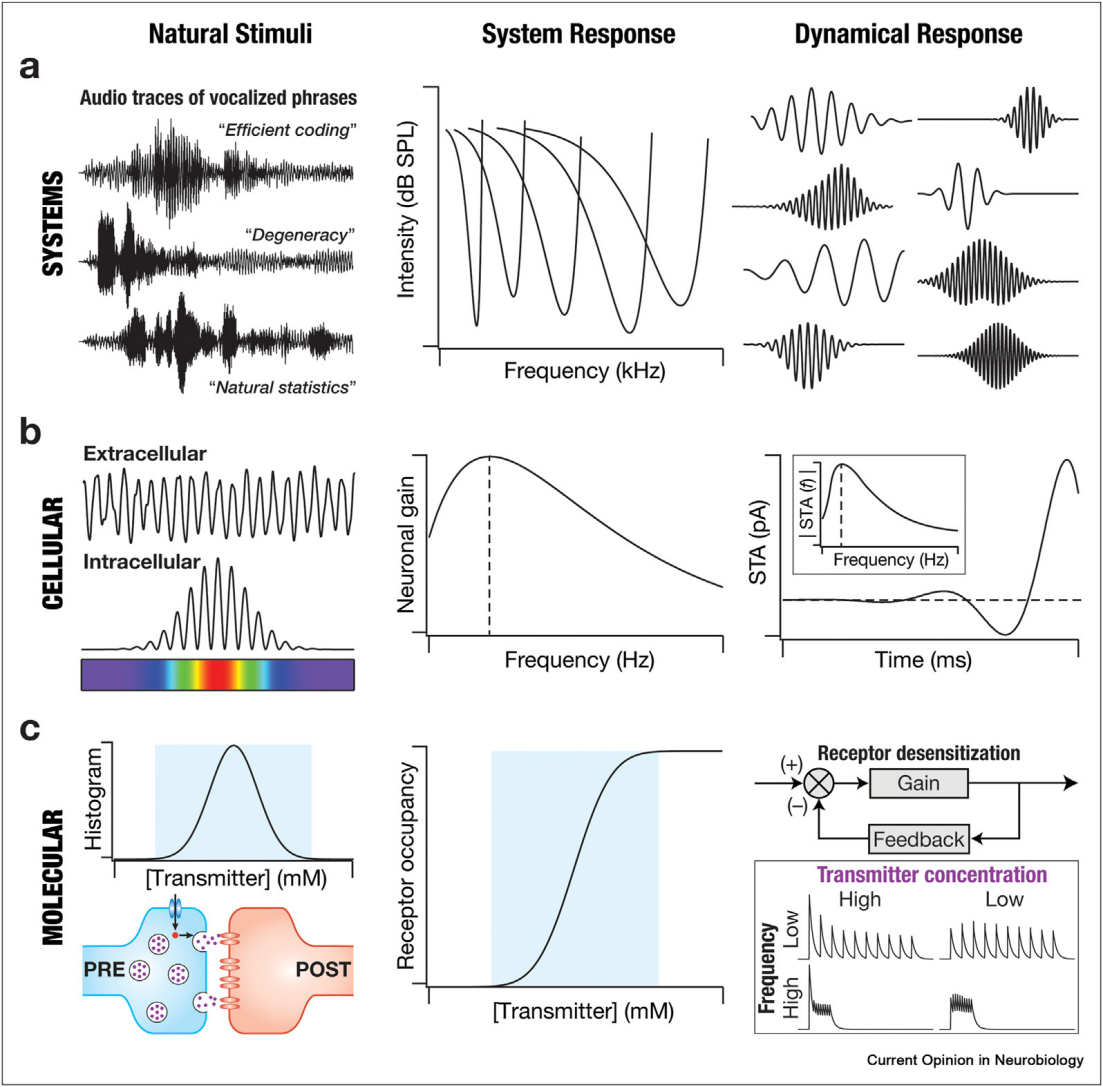
Efficient information coding across different scales of analysis. (a) Systems scale efficient coding. *Left*, audio waveforms depicting human vocalization of the phrases *‘efficient coding’*, *‘degeneracy’*, and *‘natural statistics’* as representative examples of natural stimuli processed by the auditory system. *Center*, each of the different curves represent the response properties of different neurons in the auditory system. Plotted is the minimal intensity of auditory pure tones at different frequencies required to elicit a neuronal response. The threshold is minimum at the respective characteristic frequency for each neuron, with increases observed on either side. Different neurons respond maximally to different characteristic frequencies, together spanning the range of natural auditory stimuli. *Right*, dynamical filters that were derived from natural sound statistics matched with the response properties of auditory neurons [12,13]. **(b) Cellular scale efficient coding.** *Left*, illustration of extracellular (top) and intracellular (*bottom*) waveforms, depicting naturally occurring inputs to rodent hippocampal neurons as the animal traversed a linear arena [14,48], manifesting pronounced theta-frequency oscillations. *Center*, the response properties of neurons in the hippocampal region resemble a band-pass filter, with peak response in the theta-frequency range [53]. *Right*, dynamical filters derived as the spike-triggered average manifest theta-band characteristic frequency [58]. Inset shows the magnitude spectrum of the spike-triggered average . **(c) Molecular scale efficient coding.** *Left*, a synaptic structure showing vesicular release and postsynaptic receptors. The histogram depicts the distribution of the neurotransmitter concentration in the cleft, with the cyan rectangle covering a majority of the naturally observed concentration ranges. *Center*, the occupancy characteristics of the postsynaptic receptors are aligned with the natural statistics of transmitter concentrations (cyan rectangle), thus allowing for the efficient transfer of information. *Right*, receptors manifest desensitization, which can be interpreted as a slowly decaying negative feedback loop. In the case of multiple neurotransmitter releases, both the frequency of the releases and the neurotransmitter concentration for each release play important roles in determining the efficacy of dynamical information transfer (inset). The impact of desensitization on the responses is larger when either the frequency or the concentration is high. The natural frequency of neurotransmitter release should be aligned with the neurotransmitter concentration, the receptor occupancy statistics, and the desensitization kinetics for efficient information transfer [100,101].

Biological signals can be assessed at multiple hierarchical scales, ranging from molecular-to systems-level readouts. While the activity in a receptor population conveys a subcellular response to external stimuli involving its agonists, population activity of neurons constitutes a systems-level code of sensory stimuli. Physiology across scales could be characterized by a well-defined pair of natural stimulus and response, thereby extending the concept of natural stimulus statistics to all biological scales. Such extensions have facilitated the evaluation of EIC as a match between natural stimulus statistics and system responses across all scales, while also accounting for naturally observed dynamics of stimulus attributes [6–9,16,25–27] (Figure 1).

Exploration of EIC at the systems scale traces its origin to the path-breaking frameworks proposed by Attneave [1] and Barlow [2]. The elegant observation that the response of a neuron in the blowfly visual system matched the cumulative distribution of natural stimuli (luminance contrasts) [3–5] constituted an important step for the EIC framework. Ever since, EIC achieved through the match between neuronal response properties and natural stimulus statistics has been demonstrated across visual [7,9,11,15,28–32], auditory (Figure 1a) [12,13,33], olfactory [10,34,35], and electrosensory [36,37] modalities. Importantly, although the EIC framework was proposed from a sensory neuroscience perspective, several studies provide lines of evidence for its manifestation in brain regions implicated in cognitive functions such as spatial navigation [14,38,39], value estimation and decision making [40–42]. More generally, EIC could explain states of other parts of the nervous system involved in learning complex task paradigms, perception, and motor command execution [43–47].

Information from the external world is typically represented by action-potential firing properties of individual neurons, through changes in firing frequencies and/or the timing of action potentials. The parameters intrinsic to individual neurons (morphology, ion-channel, and synaptic distributions) critically govern their ability to generate specific patterns or rates of action potentials. Alterations to single neuron properties result in massive changes to information transfer across individual neurons, even if the afferent information impinging on their synapses remain unchanged [14,38,48,49]. For instance, changes limited to ion-channel distributions critically alter the efficiency of spatial information transfer through the rate [38] or phase [14] codes in place cells. Therefore, studies analyzing the efficiency of information transfer in neural responses to external stimuli must account for the physiology of individual neurons as a critical cog in the transformation of natural stimulus statistics to a useable dynamic range of responses [14,38,50,51].

At the neuronal scale, EIC implies a match between single-neuron response properties and the statistics of different attributes of the impinging network activity [50,52–56]. In achieving EIC, single neurons adaptively tune their intrinsic properties (including ion-channel conductances) to match their response properties to the natural statistics of dendritic inputs [50–52,55,56]. Neurons in the hippocampus receive theta-modulated inputs, which translate to strong theta-frequency oscillations in their extracellular and intracellular potentials [57] (Figure 1b). The matched response properties, involving theta-frequency band-pass filtering in the impedance profile [53] and in the spike-triggered average [54,58] along the somato–dendritic axis of hippocampal pyramidal neurons, constitute an example of neuronal-scale EIC (Figure 1b).

Extensive studies involving the EIC framework at the molecular scale [6,25–27,59–68] are driven by the recognition that information about an endogenous ligand could be efficiently transmitted by matching the receptor’s response properties to natural statistics of the ligand (Figure 1c). The stimulus is defined by the abundance and the dynamics of the ligand, and the response is either the output of the receptor or of a downstream signaling cascade involving receptor activation. A recurring theme across EIC studies at the molecular scale invokes signaling motifs [69], specifically negative feedback loops, that maximize information transfer and alleviate the problem of molecular noise [25,59,60,64,66,70].

### Degeneracy supports EIC

Degeneracy is a ubiquitous biological phenomenon involving the interactions between structurally distinct components yielding similar function [17]. The manifestation of degeneracy across all neural scales and the roles of degeneracy in achieving biological robustness are well established [17–23]. Despite this, the powerful role of degeneracy as a substrate for learning, neural coding, and concomitant homeostasis is only beginning to be explored [14,20,21,24,38,48,49,71,72].

Degeneracy in EIC could manifest as system-to-system variability or as an individual system employing distinct context-dependent routes at different instances. A simple illustration of efficient information transfer occurring with these different manifestations is visualized with human communication involving written and verbal forms of different languages, dynamical gestures, and different contexts [17]. The availability of several routes to encode, adapt, match, and respond to persistent changes in natural stimulus statistics offers unique advantages to the system in maintaining robust EIC. Specifically, consider a scenario where a certain component or route fails to perform, owing to the dynamical state of the system or the component’s engagement in a different function. Degeneracy then provides a substrate for EIC through recruitment of different components/routes to execute the same task.

Degeneracy as a substrate for simultaneously achieving both EIC and homeostasis is particularly appealing because biological systems continually adapt to noisy dynamical stimuli exhibiting context-dependent natural statistics. Specifically, as the external stimuli is continually changing, there is a need to simultaneously maintain several variables within physiologically plausible levels. The availability of disparate routes ensures that the system has several degrees of freedom to simultaneously achieve these outcomes without cross-interferences. Degeneracy also favors evolvability of efficient coding by virtue of disparate structural components adapting differently to environmental changes, together offering a substrate for adaptive innovations in achieving EIC in a perpetually changing environment [17,22].

**Figure 2:**
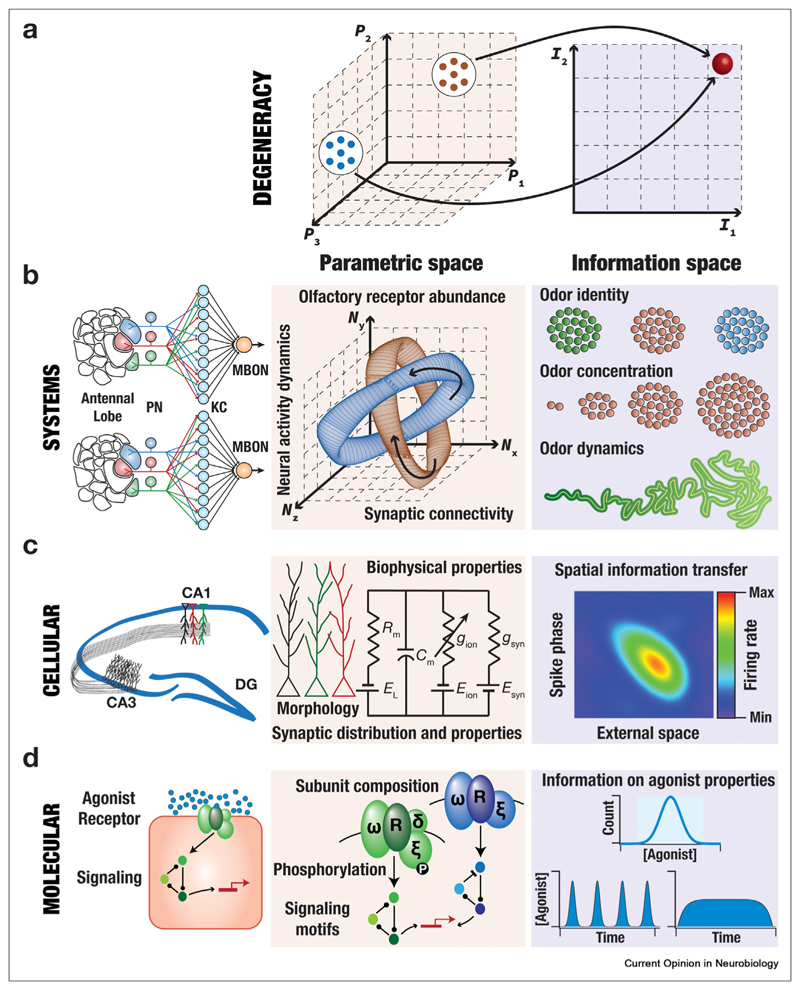
Degeneracy in the emergence of efficient information coding across scales. **(a)** Definition of degeneracy in the emergence of efficient information coding. In examples below, disparate combinations of parameters yield a similar efficiency in information transfer involving one or more stimulus attributes. **(b) Degeneracy in efficient information coding involving systems scale parameters.** *Left*, schematic representation of olfactory circuitry in *Drosophila melanogaster*. PN: projection neuron. KC: Kenyon cells. MBON: mushroom body output neuron. The PN-KC connectivity is different in the two diagrams [75,76]. The abundance of olfactory receptors is also dependent on the odor concentrations, pointing to efficient encoding that accounts for natural stimulus statistics [34,35]. *Center*, the parametric space includes the abundance of specific olfactory receptors across different animals, neural activity dynamics during odor presentation, the synaptic connectivity across different brain regions. *Right*, the information space accounts for odor identity (*top*), odor concentration (*middle*), and the spatiotemporal dynamics of odor (*bottom*). There are lines of evidence that disparate connectivity patterns between PN and KC result in stereotypic responses downstream [75,76]. Similar observations about random connectivity and stereotypic function have been made in the mammalian olfactory system as well [77,78]. **(c) Degeneracy in efficient information coding involving cellular scale parameters.** *Left*, schematic representation of the rodent hippocampus. CA1: Cornu Ammonis 1. CA3: Cornu Ammonis 3. DG: Dentate gyrus. Place cells in the CA1 manifest place-specific firing. *Center*, the parametric space includes the morphology of neurons, their biophysical properties (*e.g.* passive properties, ion-channel expression and distribution), and the distribution of synapses across the dendritic arbor [48,49]. *Right*, spatial information is transmitted through rate and phase codes. There are lines of evidence for the expression of ion-channel degeneracy in efficient spatial information transfer through rate [38] or phase [14] codes. **(d) Degeneracy in efficient information coding involving molecular scale parameters.** *Left*, illustration of a receptor on a cell surface responding to its endogenous agonist at natural concentrations through the activation of downstream signaling cascades. Shown is a scenario where receptor activation recruits a feedback motif, eventually resulting in transcriptional changes. *Center*, the parametric space includes the different subunits that the receptor is composed of, the phosphorylation status of different residues on these subunits, the properties and abundance of down-stream signaling molecules, and the signaling motifs recruited by individual receptors. Shown are two different scenarios with disparate subunit composition, phosphorylation status, and signaling motifs (positive vs. negative feedback). *Right*, the information space involves agonist concentration (top) and dynamics (bottom) and recruits the activation and dynamics of signaling molecules [26,60,79]. The bottom panel shows two graphs with phasic *versus* tonic dynamics of agonist encountered by the receptors [25]. There are lines of evidence for degeneracy in signaling dynamics involving disparate signaling molecules and pathways [65].

Degeneracy forms a reliable substrate to achieve similar information transfer efficiency through several non-unique routes. At the behavioral scale, animals are required to dynamically rely on and effectively use information from various sensory modalities to achieve functional goals such as mate attraction [73] and finding prey [74]. An outstanding example for degeneracy in systems-scale EIC involves information transfer about the identity, abundance, and dynamics of odorants by the olfactory system (Figure 2b). Through degeneracy, a parametric space spanning activity dynamics of disparate neuronal populations, random synaptic connectivity, and differential olfactory receptor abundance (Figure 2b) contributes to stereotypic functional outcomes in the olfactory system [34,35,75–78]. With reference to network-scale degeneracy in EIC, response decorrelation (reducing information redundancy is a fundamental principle governing the EIC framework; see Box 1) could be achieved through disparate forms of neural-circuit heterogeneities either individually or synergistically [71,72].

The transformation of synaptic inputs to single-neuron responses is a critical step in the cascade of transformations required for EIC of sensory stimuli by neural responses. Therefore, sensory information transfer is governed by cellular—scale parameters that mediate the input—output characteristics of individual neurons. However, efficient coding studies exploring degeneracy with reference to the impact of cellular—scale parameters on neural responses to external stimuli have been far and few. There is evidence for degeneracy in the expression of efficient spatial information transfer through rate or phase codes in hippocampal neurons (Figure 2c). These studies demonstrate that disparate combinations of cellular—scale parameters (morphology, synaptic distributions, and ion-channel expression) result in a similar efficiency in spatial information transfer [14,38].

In the molecular-scale parametric space involving receptor identity, downstream signaling motifs, post-translational modifications on receptor subunits, information typically relates to the abundance and dynamics of agonist molecules [25,26,60,79] (Figure 2d). Although the scope for the expression of degeneracy in EIC is higher at the molecular scale, given the broad parametric space, exploration has been limited. However, there are clear lines of evidence for degeneracy in signaling dynamics involving disparate signaling molecules and pathways [65].

### Heterogeneities and EIC

Heterogeneity is an inescapable reality in biological systems. Heterogeneities in the brain span molecular diversity, cell-to-cell, circuit-to-circuit, and animal-to-animal variability in characteristic properties. There are also pronounced functional distinctions in encoding and decoding strategies as well as behavioral and perceptual differences. The relationship between EIC and heterogeneities has been explored from three perspectives across scales. The simplest perspective considers heterogeneities in the parametric space as a natural consequence of the manifestation of degeneracy in the functional space of EIC. Specifically, if different individuals within a population achieve EIC through disparate parametric combinations, pronounced inter-individual heterogeneity in underlying parameters are naturally expected. The manifestation of degeneracy and heterogeneities implies that a mechanism that enhances information transfer in one individual might be detrimental in another owing to distinctions in mechanisms that yielded EIC.

**Figure 3:**
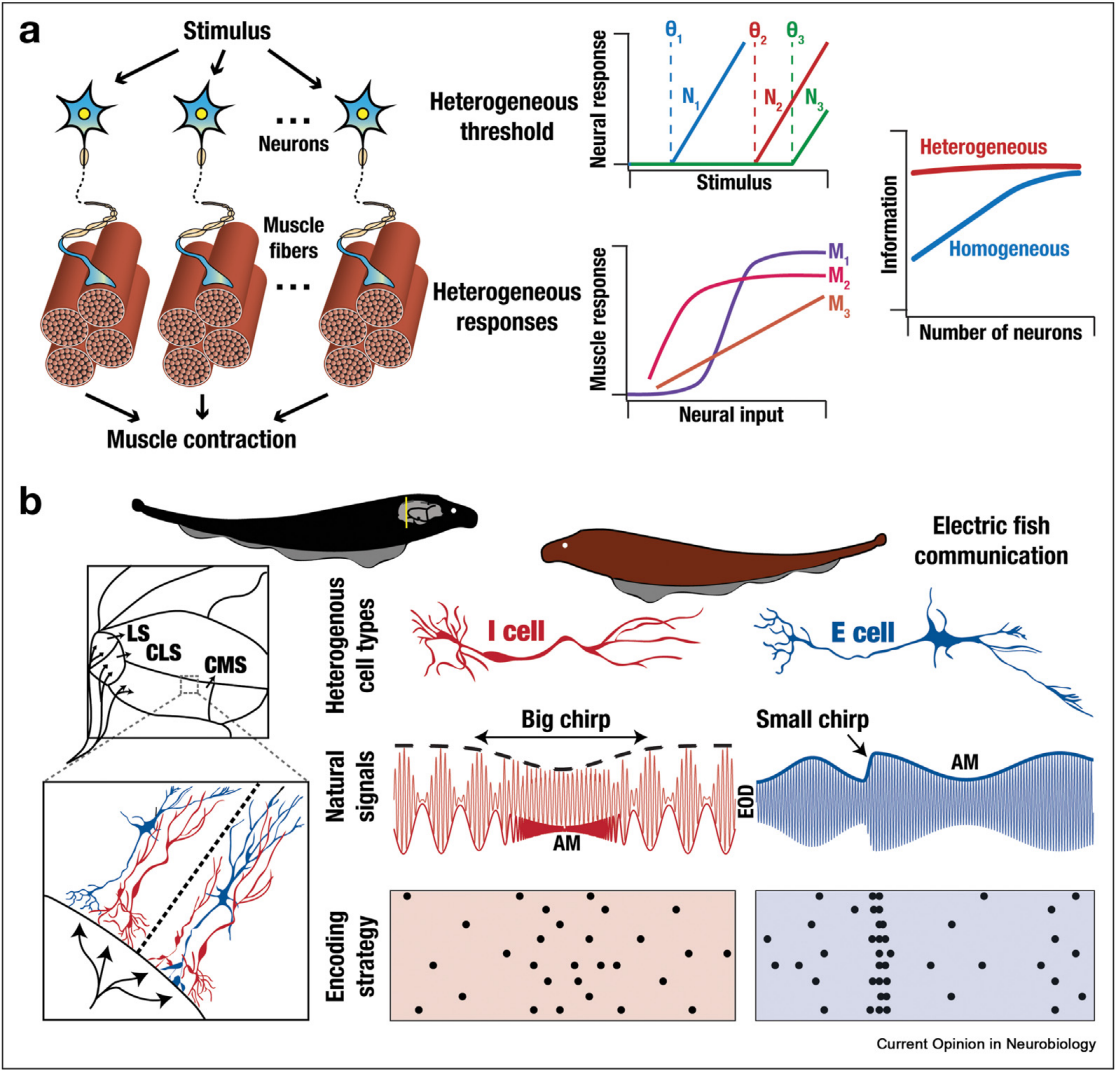
Heterogeneities and efficient information coding. **(a)** Illustration of a scenario where heterogeneity in response properties contributes to efficient coding. *Left*, schematic showing the flow of information from stimulus to neurons to muscle fibers, eventually resulting in muscle contraction. *Center*, the top panel shows heterogeneities in neural responses, with different neurons showing different threshold values (θ_i_). The bottom panel illustrates heterogeneities in muscle output in response to neural inputs. *Right*, cell-to-cell variability across muscle fibers results in efficient information coding by enhancing representation for a larger stimulus range [44,82]. **(b)** Illustration of a scenario where the implementation of efficient coding can explain the emergence of heterogeneity in response to properties of different cells within the same system. *Left*, schematic of weakly electric fish (*Apteronotus leptorhynchus*) showing the section (yellow line) of the brain (gray) expanded below. Shown in the expanded version are three segments — centromedial (CMS), centrolateral (CLS), and lateral (LS) — of the electrosensory lateral line lobe in the brain. Representative E (blue) and I (red) cells, two distinct classes of pyramidal cells in the electrosensory lateral line lobe, are shown in the further zoomed version below. Derived from Hoffman and Chacron [36]. *Right*: *Top*, the E *versus* I heterogeneity within the pyramidal neuron population has been implicated in sparse neural coding, whereby E- and I-cells selectively respond to conductors *versus* non-conductors in the fish**’**s natural environment, respectively. *Middle*, big (red) and small (blue) chirp signals consequent to natural interactions associated with courtship and aggressive encounters with other fishes, respectively. Shown are the amplitude modulation (AM) envelope and the electric organ discharge (EOD) for both signals. The second order envelope is also shown for the big chirp signal (dashed black line). *Bottom*, I cells prefer big chirps and respond with increased firing rates (left), whereas E cells preferentially respond to small chirps with spike bursts (right). Here, the emergence of heterogeneity in the pyramidal cell population is an effect of different sub-populations matching different aspects of the stimulus statistics toward eliciting preferential responses to different signals.

A second perspective, through the lens of reducing information redundancy [31,50,71,80,81], provides evidence that heterogeneities favor the emergence of EIC by forming a substrate for individual units to encode distinct information about afferent stimuli (Box 1). Such active recruitment of heterogeneities allows decorrelated responses across units, yielding efficient and non-redundant distribution of information. A recent study reported that intercellular heterogeneities within skeletal muscle fibers enhance information transmission (Figure 3a) owing to such response diversity [82]. Biophysical heterogeneities across cells in the cochlear nucleus [83] and inferior colliculus [84] have been shown to improve population coding of auditory stimuli. Different forms of neural-circuit heterogeneities have been shown to enhance decorrelation or network information transmission through either temporal or rate coding strategies [71,72,85,86].

A third perspective explains response heterogeneities to be consequent to EIC, whereby different sub-populations match their response properties to respective natural stimulus statistics. As a broad example, the response profiles of auditory and visual neurons are critically dependent on the natural stimulus statistics they encode [87]. The functional heterogeneity involving two kinds of pyramidal neurons (E *vs*. I cells) in the electrosensory lobe of electric fishes (Figure 3b) are related to their preferential responses to different afferent stimuli associated with aggressive and courtship communication signals [80]. More subtly, heterogeneity across I-cells has been shown to govern efficient encoding of the quality of the courtship signals [80]. In mice, the dorsal and ventral retinal circuits manifest differential color-opponency, by virtue of distinct spectral characteristics of the cones that form their respective natural stimulus statistics [30].

### Adaptation and stability

**Figure 4:**
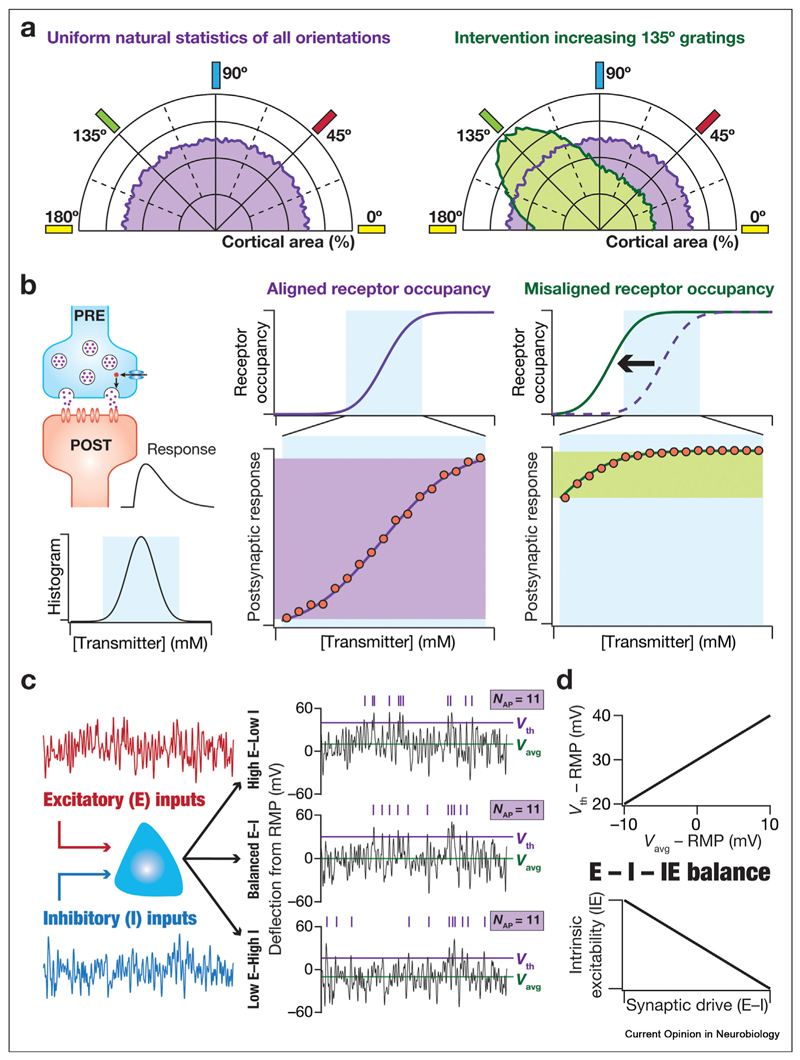
Plasticity and homeostasis in efficient information coding. **(a)** Illustrative of a scenario where a change in natural statistics triggers the change in response properties, maintaining efficient information coding. *Left*: natural visual stimulus is endowed with all orientations. The cortical area is allocated uniformly across all orientations (purple). *Right*: an artificial intervention, involving rearing of animals in a striped environment, enhances the prevalence of inputs oriented at 135°. The cortical area allocated for 135° is higher (green) than other orientations [89]. Observational approaches to efficient coding assess the relationship between natural stimulus statistics and response characteristics to unveil a match. Interventional approaches, such as the example provided here, add further evidence for efficient coding by demonstrating that targeted manipulations to stimulus statistics introduce matching changes in response properties. **(b)** Illustration of a scenario where efficient information coding is hampered when response properties change, but natural statistics remain unaltered. *Left*: a synaptic structure showing vesicular release and the postsynaptic response depicted as an electrical deflection. The histogram depicts the distribution of the neurotransmitter concentration in the cleft, with the cyan rectangle covering a majority of the naturally observed concentration ranges. *Center*: the occupancy characteristics of the postsynaptic receptors (purple line) are aligned with the natural statistics of transmitter concentrations (cyan rectangle). The postsynaptic response (amplitude of electrical deflection; red circles) spans the entire dynamic range (purple rectangle) thus allowing for the efficient transfer of information conveyed by the naturally occurring transmitter concentrations. *Right*: a leftward shift in the occupancy characteristics of the postsynaptic receptors (from purple to green) results in misalignment of the transmitter concentration distribution (cyan rectangle) and the occupancy profile (green). Consequently, the postsynaptic response (red dots) to naturally occurring neurotransmitter concentration starts at high values, yielding saturating responses for a large range of natural concentrations. The dynamic range of responses (green rectangle) is limited under such misalignment. The bottom plots of the center and the right panels also depict zoomed versions of the receptor occupancy profiles (purple and green lines, respectively) from the top plots. A linear relationship between postsynaptic response and receptor occupancy is assumed here but that relationship could change in response to changes in neuronal morphology or intrinsic properties. Efficient information transfer could be hampered because of misalignment in this relationship as well. There are examples of hampered efficient coding of agonist concentration with misalignment in receptor characteristics and downstream signaling characteristics [59]. **(c)** *Left*, illustration of a neuron (cyan) receiving excitatory (E) and inhibitory (I) inputs. *Right*, illustration of voltage responses, depicted as deflections from resting membrane potential (RMP), for three distinct scenarios for the proportion of E versus I inputs to the neuron. *Top*, High E–Low I: V_th_ = RMP+40 mV, V_avg_ = RMP+10 mV. *Middle*, Balanced E–1: V_th_ = RMP+30 mV, V_avg_ = RMP. *Bottom*, Low E-High I, action potential threshold, V_th_ = RMP+20 mV, average response voltage, V_avg_ = RMP-10 mV. The purple ticks above each panel depict spike times based on the voltage response crossing the respective V_th_. Note that the number of spikes elicited in each of these three configurations was the same (*N*_AP_ = 11) because of concomitant changes in the E-I ratio and V_th_. **(d)** The balance between excitation-inhibition and intrinsic excitability (E–1–IE) is critical for the emergence of homeostasis and efficient coding. Top: plot of the threshold voltage (*V*t_h_) *versus* the average response voltage (V_avg_) derived from the three cases depicted in panel **c**, each eliciting the same number of action potentials. *Bottom*: a generalized depiction of the plot from the top panel showing intrinsic excitability *versus* synaptic drive. As a higher *V*_th_ translates to lower excitability, this generalized plot illustrates the requirement of E–1–IE balance in maintaining concomitant homeostasis and efficient coding [14].

Correlational lines of evidence for EIC are typically based on a match between information-maximizing filters that are derived from natural stimulus statistics and experimentally observed response characteristics [9,11, 12,29,33–35]. On the other hand, studies that causally test the efficient coding hypothesis recruit interventional methods that perturb the natural stimulus statistics to assess if responses adapt to match the altered stimulus statistics. Such systematic manipulations to natural stimulus statistics have been performed across sensory modalities and have provided evidence supporting EIC. In the visual system, monocular deprivation or altered distributions of stimulus orientations (Figure 4a) were employed to demonstrate adaptations matching altered statistics [88,89]. Monaural deprivations or exposure to specific tone frequencies resulted in matching alterations in the organization of auditory cortex [90–93]. Nostril closure or altered odorant exposures resulted in changes in the abundance and properties of the olfactory neurons [34,35,94]. Importantly, specific plasticity and neuro-modulatory mechanisms have been identified to mediate adaptive strategies that lead to efficient codes [91,95,96]. Such adaptation has also been addressed from a dynamical perspective with the continual matching of response properties to time-varying inputs [15,16].

In contrast to these examples where system responses showed adaptive plasticity to altered natural stimulus statistics, there could be changes in system responses that are not triggered by changes in environmental stimulus distributions. For instance, efficient coding of natural ligand abundance is achieved through negative feedback mechanisms that match receptor occupancy and a downstream response [59]. Perturbations to the signaling cascades without change in ligand distributions introduce misalignment of the dose—response relationship, resulting in response saturation and noise amplification [59,79]. Similarly, perturbations to postsynaptic receptor identity or input—output characteristics, without changes in transmitter statistics, would yield misalignment between the natural stimulus statistics of transmitter abundance and receptor occupancy. Such misalignments of the useful response range result in the saturation of postsynaptic responses and a loss of information about transmitter abundance (Figure 4b). Together, it is essential to assess perturbations (induced by activity, neuromodulation, or pathology) to response properties that hamper EIC by misalignment of response characteristics with natural stimulus distributions.

Continual adaptations to natural stimulus statistics need to sustain concomitant stability to avoid pathological adaptations and maintain homeostasis. Different measures for homeostasis have been proposed across scales, including excitation-inhibition (E–I) balance at the network scale, firing rate and voltage levels at the cellular scale, and calcium levels at the molecular scale [97–99]. A unified metric that accounts for E–I balance and intrinsic excitability (IE, which governs firing rates and voltage levels for a specific synaptic drive) is the E—I–IE balance [14]. Homeostasis of firing rate can be achieved if the E—I ratio is counterbalanced by changes in action potential threshold that governs IE (Figure 4c). Importantly, it has been shown that EIC is achieved when there is a counterbalancing relationship between the overall synaptic drive (E–I) and neuronal gain (IE) [14]. Finally, strong lines of evidence for degeneracy in homeostatic maintenance [21,24,97], together with degeneracy in EIC explored earlier, suggest degeneracy as a efficacious substrate for achieving efficient encoding and concurrent homeostasis [14,38,71].

## Conclusions

Our synthesis spanning several species and multiple scales demonstrates EIC as a generalized biological principle. We argued that biological complexity and ensuing degeneracy are central cogs in the concurrent emergence of EIC and homeostasis. We emphasized the critical roles for parametric heterogeneities as well as dynamics (associated with the stimulus and the response spaces) in improving information transfer. We postulate the interplay between EIC and homeostasis as a universal repeating motif whose balance governs biological systems across scales. The recognition of the ubiquitous nature of these governing principles and explorations focused on degeneracy as a substrate for their concurrent emergence would pave the way for deducing the beneficiary roles of complexity across all biological systems [17].

## Box 1 Definitions of important terms.

### Efficient information coding

An overarching principle that states that systems can efficiently process their inputs by matching their response properties to their natural stimulus statistics, together maximizing information transfer [1–16]. Although certain definitions of efficient coding also encompass energy efficiency, our focus here is exclusively on information transfer efficiency.

### Complex system

A system in which smaller parts are functionally segregated or differentiated across a diversity of functions but shows increasing degrees of functional integration when more and more of its parts interact [17].

### Degeneracy

The ability of elements that are structurally different to perform the same function or yield the same output [17]. Degeneracy has been shown to be prevalent across all scales of neural systems [18–24].

### Homeostasis

Homeostasis is a self-regulating process by which biological systems tend to maintain stability while adjusting to conditions that are optimal for survival.

### Structural redundancy

*Structural redundancy* in systems refers to *structurally identical* elements performing the same functions to ensure fail safe operation wherein an identical substitute executes the function in an identical manner when the original component is unable to. Structural redundancy is fundamentally different from degeneracy, where structurally distinct elements are involved in executing the same function. While redundancy can exist even in simple systems, degeneracy is defined to be emergent in complex systems as a consequence of *functional integration* of *functionally segregated* structures/subsystems. Structural redundancy does not offer several advantages of degeneracy, including evolvability and robustness to component-specific perturbations [17,20,22].

### Information redundancy

Information redundancy in encoding refers to information being redundantly represented by different neurons or networks of neurons [1,2,5]. Information redundancy hampers efficiency, thus making redundancy reduction as an important aspect of achieving EIC.
